# Changes of Dental Implant Surgery-Related Anxiety and Pain with Respect to ASA-Physical Status

**DOI:** 10.3390/jcm13226686

**Published:** 2024-11-07

**Authors:** Alper Sağlanmak, Volkan Arısan

**Affiliations:** Department of Oral Implantology, Faculty of Dentistry, Istanbul University, Fatih 34093, Türkiye; varisan@istanbul.edu.tr

**Keywords:** dental anxiety, pain perception, dental implants

## Abstract

**Background/Objectives:** Rehabilitation of missing teeth with dental implants is a strong trigger of dental anxiety. The sympathetic response caused by anxiety can lead to cardiovascular and cerebrovascular incidents, especially in patients at systemic risk (ASA Physical Status-II and ASA Physical Status-III). Dental anxiety can affect physical health by activating the sympathetic response, and the patient’s physical health status may also affect dental anxiety. The aim of this study was to analyze the factors that may reduce anxiety and pain, considering the patient’s physical health status according to American Society of Anesthesiologists (ASA-PS). **Methods:** A total of 562 implants were placed under local anesthesia in 201 patients with ASA PS-I (healthy) and ASA PS-II and III (comorbid). The effect of patient-, dentist-, and surgery-related variables on dental anxiety and pain perception were evaluated. Modified Corah Dental Anxiety Scale and Numerical Rating Scale for Pain scores were measured and recorded preoperatively (T0) and 1 week postoperatively (T1). The effects of the associated variables were analyzed using binary logistic regression and non-parametric tests (*p* < 0.05). **Results:** Age (OR = 1.089), gender (OR = 6.493), ASA-PS (OR = 13.912), and the number of placed implants (OR = 0.807) were significantly associated with reduction in dental anxiety. There were statistically significant differences between the study groups in terms of mDAS score reduction (*p* = 0.028). **Conclusions:** ASA-PS, gender and the number of placed implants affected the anxiety of the patients. Age and number of implants seem to be variables with a relative influence that depends on other factors.

## 1. Introduction

Although the current advancements in technology have alleviated some of the fear associated with dental implant procedures, odontophobia—dental anxiety or fear—is still observed in a segment of society and appears to be a speculative topic [1]. Dental phobia has higher prevalence (~12%) than many other phobia subtypes in the general population [2]. Dental anxiety was first defined by Corah in 1969 as “the stress shown by the patient during a dental visit” [3]. Subsequently, the concept was subjected to repeated evaluation and redefinition for different age groups and treatment approaches. A variety of measurement techniques have been proposed to assess dental anxiety, including behavioral ratings, psychometric scales, physiological measures, and projective techniques [4,5]. The scales utilized for adults are usually the Corah Dental Anxiety Scale (DAS), Dental Fear Scale (DFS) [6], and Modified Dental Anxiety Scale (mDAS) [7]. In addition to the aforementioned methods, a variety of scales may be employed for this purpose, including the Venham Picture Test [8] and those measuring State-Trait Anxiety [9], which has been acknowledged as more comprehensive. Anxiety scales incorporating an innovative artificial intelligence-based algorithm have been developed for assessing dental anxiety in a more objective manner [10].

A wide range of psychological factors are responsible for dental anxiety and dental behavior management problems [4]. This problem poses the risk of partial or complete abandonment of the treatment or deterioration of the cooperation between dentist and the patient and also has the capacity to reduce the success of the treatment applied [11]. Recognition and awareness of the situations that may lead to anxiety and odontophobia is crucial in overcoming clinical challenges during treatment. Social environment, past traumatic experiences, and speculation about the treatment protocol are all significant contributors to dental anxiety [12]. Furthermore, a missing tooth [13] and replacement with a dental implant has been reported to be a stronger anxiety stimulus than pain perception [14]. Given the complex nature of dental anxiety, there has always been a keen interest in investigating the impact of assorted variables and techniques. Dental anxiety and pain in implant surgery may be attributed to a number of factors such as age, gender, type of surgery [15], surgical complexity and number of implants [16], American Society of Anesthesiologists Physical Status Classification (ASA-PS) [17], prior surgical intervention, experience of the surgeon [18], etc. It is also important to consider other factors that may contribute to the development of dental anxiety, such as family history [19], the presence of a gag reflex, and emetophobia [20]. In addition, the patient personality is also a significant factor [21], and it is recommended that this be eliminated by examining the reduction in dental anxiety from preoperatively (T0) to 1 week postoperatively (T1). Dental anxiety, stemming from the reasons listed above and more, can make the dentist’s work challenging during implant surgery. This results in the treatment being more complex and difficult and increases chairside time. This may also prolong the procedures, increase the postoperative swelling and pain [16,22], and reduce patients’ satisfaction [23], and may even damage dental implants by initiating clenching and grinding [24].

The number of patients with a status requiring either simple or complex dental implant surgery is rising in parallel with the increase in life expectancy [25]. The utilization of implants has been on the increase, particularly among the elderly population [26]. It is a common occurrence for elderly patients to present with one or more comorbidities [27]. The risk of such complications is further amplified if the individual has a dental fear or anxiety above a certain level, which can precipitate vasovagal syncope, tachycardia, increased heart rate, hypertension, and even cardiovascular and cerebrovascular accidents, particularly in patients with systemic risk (ASA PS-II, ASA PS-III) [17]. The American Society of Anesthesiologists has presented a series of examples to explain the systemic risk. Classes I, II, and III define the patient’s preoperative ‘initial physical state’ (Appendix A; American Society of Anesthesiologists Physical Health Status Classification), while Class IV includes life-threatening extreme systemic conditions and Classes V and VI are reserved for patients scheduled for emergency surgery [28]. Information on dental anxiety and patient vital signs during dental procedures is scarce [29]. Therefore, assessing comorbid (ASA-PS II and III) patients in relation to their dental anxiety and aiming for reduction in anxiety and pain can be hypothesized to prevent cardiovascular and cerebrovascular complications. Dentists should consider ASA-PS when making preoperative decisions and determining the use of analgesics and anesthesia, as it may influence anxiety and pain.

The aim of this study is to investigate the relationship between anxiety and pain in healthy (ASA-PS I) and comorbid (ASA-PS II and III) patients undergoing dental implant treatment, with factors related to the patient, dentist, and surgery. The null hypothesis of the study is that patients with ASA-PS II and III present with comparable levels of dental anxiety and pain perception scores to those with ASA PS I.

## 2. Materials and Methods

### 2.1. Sample Size Calculation

The prevalence of dental anxiety was taken as 10% as in the study by Doerr et al. [30]. With a prevalence difference threshold of 15%, set at an alpha value of 0.05 and a power of 0.80, 201 patients are required (*p* < 0.05). Software was used for the calculation (G Power version 3.1, Dusseldorf, Germany). 

### 2.2. Allocation of the Patients

This study was approved by the Ethical Committee of the Faculty of Dentistry, Istanbul University (No: 2024/16), and was conducted in accordance with the Helsinki Declaration (as revised in 2013) [31]. Patients who applied to Istanbul University Faculty of Dentistry, Department of Oral Implantology, for dental implant treatment between February 2024 and August 2024 were considered for screening. After a clinical and radiological examination, patients who were suitable for implant treatment were considered for inclusion. The study outline was explained, and approving patients were enrolled by written consent. A total of 211 partially and completely edentulous patients were investigated by a questionnaire about dental anxiety (mDAS) and pain perception (NRS) prior to surgery (T0) and 1-week post-op (T1). Patient allocation was ended with the completion of the pre-determined sample size of 201.

### 2.3. Inclusion and Exclusion Criteria

The following inclusion criteria were defined for patient selection:

Patients who were (1) ASA PS-I (healthy) or II or III (comorbid); (2) locally and systemically eligible for dental implant treatment with local anesthesia; (3) 18 years of age or older; (4) in need of straightforward, advanced, or complex surgery (SAC) [32]; (5) willing, able to read, understand, and complete the questionnaire form.

The exclusion criteria were as follows:

(1) High-risk patients (ASA IV); (2) those refusing to participate; (3) patients with a treatment need other than the conventional frame of the SAC.

### 2.4. Demographic Data and Variables

The following variables were analyzed:

(1) Patient’s age, (2) gender, (3) systemic status (ASA-PS I/II and III), (4) education (university and above/below), (5) prior experience of implant surgery (yes/no), (6) dentist’s experience (specialist/resident), (7) number of placed implants in the same session, (8) surgery time (minutes), and (9) surgical complexity (straightforward/advanced/complex).

### 2.5. Study Design

As the reliability and validity of the mDAS in the Turkish population have been previously established [33], the Turkish translated version of the mDAS was used in this study (e.g., Appendix B: Modified Dental Anxiety Scale Questionnaire). The assessment of pain perception was conducted utilizing the NRS [34], which was selected for its simplicity and uniformity of format [35] (e.g., Appendix C: Numerical Rating Scale for Pain Questionnaire). A total of five questions were posed to patients on the anxiety scale, with each question requiring a single response from the patient. The options are scored from 1 to 5. The minimum score is 5 and the maximum total score is 25. Patients with scores between 5–10 were defined as low anxious, 11–18 as moderately anxious and 19 or above as highly dentally anxious or phobic [33]. Patients were directed to rate NRS to estimate the potential pain perception they might experience at baseline. A rating of 0 represents no pain, 1 to 3 mild pain, 4 to 6 moderate pain, and 7 to 10 severe pain.

A questionnaire form developed to assess dental anxiety and pain perception was prepared. Prior to surgery (T0), patients were seated at the waiting area for a period of 30 min and dental anxiety and pain perception were measured by questionnaires based on the aforementioned mDAS and NRS indexes.

Surgical operations were performed under local anesthesia and aseptic conditions by one resident (HB) and one specialist (AS) at the Department of Oral Implantology, Faculty of dentistry, Istanbul University, Turkey. The resident has undergone a four-year post-graduate program, while the specialist has accrued ten years of experience. The allocation of patients among the specialist and resident was based on a combination of patient’s preference and availability of an appointment. Surgical procedures consisted of implants (*n* = 144) and GBR (guided bone regeneration) (*n =* 57). GBR were accomplished by the use of a particulate graft (Bio-Oss^®^, Geistlich Pharma AG, Wolhousen, Switzerland) and a resorbable membrane (Bio-Gide^®^, Geistlich Pharma AG, Wolhousen, Switzerland). Implant surgery was performed in line with the manufacturer’s instructions, either submerged or non-submerged via a healing abutment. A supplementary form was prepared for surgery-related factors (surgical complexity, number of placed implants, and surgery time (min)). The surgery time was recorded by the dental assistant from the first incision to the end of the final stich. The surgical complexity of the procedures was classified according to the SAC system, which categorizes procedures as straightforward, advanced, or complex. All complications and events during and after the surgery were recorded.

Following surgery, patients were prescribed antibiotics (amoxicillin 875 mg twice daily for 5 days; Augmentin^®^, Glaxo-Smith-Kline, İstanbul, Türkiye), antiseptic mouthwash (chlorhexidine gluconate 0.12%; benzydamine hydrochloride 0.15%, Andorex^®^, Humanis, İstanbul, Türkiye), and analgesics (50 mg dexketoprofen trometamol; Dex-Forte^®^ 50; Neutec, İstanbul, Türkiye) for a period of 5 days. The following week, patients were recalled and a new questionnaire was administered, comprising the mDAS and NRS.

### 2.6. Statistical Analysis

The normality of the distribution of the data was assessed using the Kolmogorov–Smirnov test. The chi-square test and Mann–Whitney-U *t*-test were used to assess the similarity of baseline variables between groups. MDAS and NRS score reduction in time (T0–T1) was evaluated with the Wilcoxon signed rank test. mDAS scores were compared among study groups. A logistic regression model was used to evaluate the effect of variables. The backward elimination method with Wald statistics was utilized to identify important variables. The fit between the actual and predicted values of the dependent variable was assessed using the Hosmer–Lemeshow test. The efficiency of the model was measured using Nagelkerke R^2^. Odds ratios (ORs) and parameter estimates (β) were calculated for all variables in the regression model. *p* < 0.05 was considered statistically significant. All statistical analyses were performed using SPSS^®^ (version 29.0.20.0) for Mac (IBM Corporation, Armonk, NY, USA, 2024). The Strengthening Reporting of Observational Studies in Epidemiology Checklist (STROBE) was employed for the preparation of this manuscript [36].

## 3. Results

**Figure 1:**
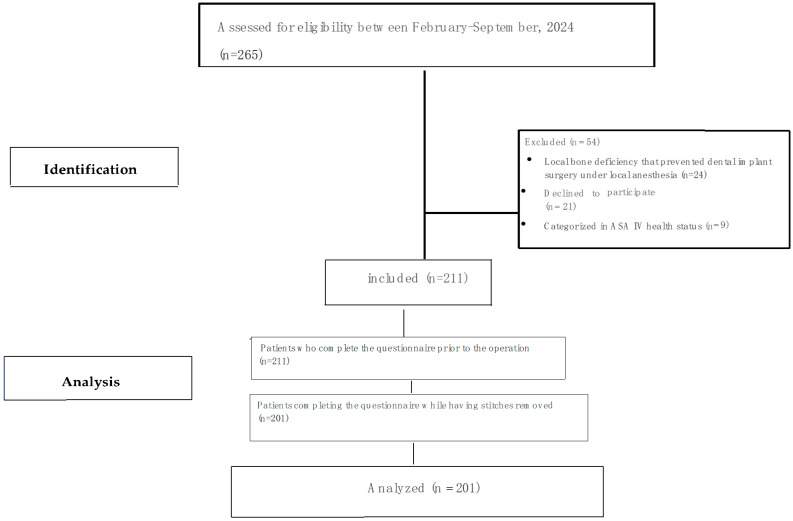
Study flow chart of the study population.

Two hundred and sixty-five patients who applied to Istanbul University Faculty of Dentistry, Department of Oral Implantology, for dental implant treatment were examined between February 2024 and September 2024. A total of 24 patients were excluded due to local bone deficiency that prevented dental implant surgery under local anesthesia. Furthermore, 9 patients were excluded due to being categorized in ASA IV health status. Of the remaining 232 patients, 21 refused to participate in the study and a total of 211 patients were included. Ten questionnaires were excluded from the analysis on the grounds that they had not been completed. Finally, 201 patients and 562 implants were included for analysis (Figure 1. Study flow chart).

Descriptive data for the baseline variables are presented in Table 1. A total of 201 patients with a mean age of 45.92 ± 11.65 years received 562 screw type titanium implants both in the maxilla and mandible. Any complications were observed during a 1 week period. The patient group consisted of 102 women and 99 men. A total of 89 patients had attained a university degree or higher level of education, while 105 patients had obtained qualifications below this level. A total of 130 patients were ASA-PS I (65% healthy), whereas 71 were ASA-PS II and III (35% comorbid). The baseline anxiety status of the patients was analyzed, with 72.7% exhibiting low anxiety, 17.4% demonstrating moderate anxiety (11–18), and only 9.9% categorized as phobic (>19). A total of 201 patients were included in the study, of whom 145 received multiple implants (4.11 ± 2.34) and 56 received single implants. Mean surgical time was 60.74 ± 44.59 min. A total of 73% of the surgical procedures were performed by resident and 27% by specialist. A total of 60% of the patients had dental implant surgery before. In accordance with the degree of surgical complexity, 60% of the cases were classified as straightforward (*n* = 120), while 40% were categorized as advanced or complex (*n* = 81). No complications were encountered during surgical procedures.

**Figure 2:**
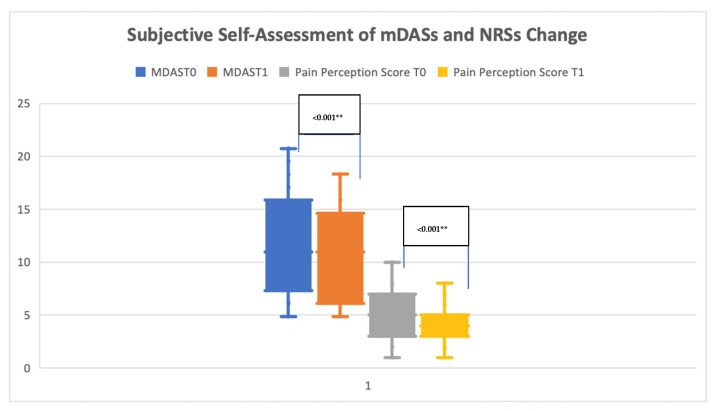
Box-plot graphic of mDASs and NRSs change. ** *p* < 0.01.

The mean baseline (T0) mDAS score for the patient cohort was 11.57 ± 4.85 (median: 11/IQR: 7), while T1 was 10.26 ± 3.99 (median: 11/IQR: 9). The change between the two-time intervals was found to be statistically significant (Wilcoxon signed-rank test *p* < 0.001). NRS scores were 5.26 ± 2.30 (median: 5/IQR: 4) and 3.88 ± 1.66 (median: 4/IQR: 2), respectively. The change between the two-time intervals was found to statistically significant (Wilcoxon signed-rank test *p* < 0.001) (Table 2, Figure 2). The distribution of mDAS and NRS scores over time stratified by study groups (healthy and comorbid) is presented in Table 3. The non-parametric test revealed a statistically significant reduction in mDAS scores among the study groups (Mann–Whitney-U test *p* = 0.028), while no statistically significant reduction was observed in terms of NRS scores (Mann–Whitney-U test *p* = 0.573).

The backwards elimination technique was used to create a significant regression model with the variables. Despite extensive analysis, no significant model could be established for the reduction in the NRS score. However, a four-variable model was identified for the **‘Dental Anxiety Reduction’**. Prior experience of implant surgery, dentist experience, surgery time, and surgical complexity were excluded and a regression model including **‘Age’**, **‘Gender’**, **‘ASA-PS’**, and **‘Number of placed implants’** was constructed. Logistic regression analysis revealed that ‘Age; (OR: 1.089, *p*: 0.003), ‘Gender’ (OR: 6.493, *p* < 0.001), ASA-PS (OR: 13.912, *p* < 0.001), and ‘Number of placed implants’ (OR: 0.807, *p*: 0.051) were significantly associated with the dental anxiety reduction. An increase in ‘Age’ and decrease in ‘Number of placed implants’ was associated with an increase in the probability of dental anxiety reduction. The ‘Gender’ being male and being in the ‘ASA-PS-I’ (healthy) increased the probability of anxiety reduction up to 6.493 and 13.912 times, respectively (Table 4).

Given the significance of gender in the regression model, a non-parametric test was also conducted to assess potential differences between genders (Table 5). Considering the gender variable, men showed significantly more MDAS score reduction (Mann–Whitney-U test *p* < 0.001). NRS scores revealed a significant difference only at T1 (Mann–Whitney-U test 0.023).

## 4. Discussion

In this prospective observational study, factors affecting anxiety and pain related to dental implant experience were investigated. A comprehensive set of variables was assessed in a relatively large group of patients, allowing rigorous comparisons between groups. Both groups showed a decrease in mDAS and NRS scores. However, this difference reached statistical significance solely in regard to for mDAS scores. The findings indicated that ASA-PS, age, gender, and number of placed implants had a notable impact on the reduction in dental anxiety.

The impact of dental implant surgery on patient-reported outcome measures has been the subject of extensive investigation, with the influence of various covariates being a key area of focus [37,38]. Several other factors including socioeconomic status, educational level, and nationality have been identified as potential correlates of dental anxiety [39,40]. A variety of techniques have been employed to address this issue, including the utilization of verbal-visual information [41], virtual reality [2], mindfulness therapy [9], music [42], conscious sedation [43], and guided meditation [44].

There are only a few studies with a large number of patients examining the changes in dental anxiety and pain perception in terms of associated indicators [15]. Furthermore, the relationship between anxiety and ASA-PS reveals a new factor involved in dental anxiety. It is established that acute stress and anxiety during dental surgery can precipitate cardiovascular and cerebrovascular accidents [45]. Nevertheless, the question of how a patient with medical comorbidities can develop anxiety about implant surgery is a topic that is still open to debate. The relationship between these factors has been the subject of only a limited number of studies [17,46]. As reported by Turer et al. [9], “There may well be more confounding factors that explain the variance in anxiety and hemodynamic parameters (such as age, gender and systemic health) than those found in this study”, which underscores the significance of the findings presented in this study. While the ASA PS-II and III are not contraindicated for the placement of implant-supported prostheses [47], an ASA IV classification denotes a situation that is potentially life-threatening [48]. Therefore, patients with ASA IV were excluded from the study.

Dental anxiety scales have been developed to measure the level of stress experienced by patients. The first scale employed was the Dental Anxiety Scale (DAS), which was developed by Corah in 1978 [49]. Although the DAS is the most widely used and validated test for dental anxiety, we preferred to utilize the mDAS [7], which includes a question about local anesthesia and has been subjected to a reliability and validity study in Turkish version [50]. Furthermore, more specialized state and trait anxiety scales (STAI-T and STAI-S) have also been developed [51], but their use in dentistry has been limited as it would be difficult for the patient to self-report.

The mean baseline (T0) scores according to the Modifying Dental Anxiety Scale Score (mDASs) of the patients was 11.57 ± 4.85 while that at T1 was 10.26 ± 3.99. The change between the two-time intervals was found to be statistically significant. Similarly, Turer et al. [9] conducted a comparative analysis of anxiety scores between implant patients and identified comparable baseline scores (12.9 ± 2.8) and significant reduction in anxiety. In the study by Saba et al. [52], a relatively low mDASs median (9) was observed. It should be noted that this questionnaire was not administered prior to the initiation of any specific treatment but rather during a general dental examination. Severe anxiety/phobia was observed in only 9.9% of patients in the present study, which is in line with the previous literature [52,53]. Yet the prevalence of anxiety exhibits considerable variation across different countries, gender, and age groups [54,55,56]. Furthermore, the advent of novel techniques (mindfulness, meditation, auditory distraction) for the alleviation of anxiety has resulted in a reduction in the prevalence of anxiety disorders [9,57,58,59].

Analysis of the patient cohort revealed no statistically significant relationship between the baseline anxiety scores and the ASA-PS classification. In contrary, Facco et al. found a significant relationship between ASA-PS and baseline anxiety [17]. Similarly, Xue et al. observed increased anxiety scores before tooth extraction in patients with cardiovascular disease [60]. The discrepancy in results may be attributed to the relatively limited number of subjects included in the study and the methodology employed. Furthermore, the influence of the physician (resident/expert) performing the procedure on the level of dental anxiety experienced by the subjects at baseline may have contributed to this observed difference. It seems reasonable to posit that dental experts are better equipped to analyze and respond to dental anxiety [61]. The study revealed that the experience of the physician did not have a statistically significant impact on the dental anxiety reduction in the patients. However, it can be postulated that extended treatment times for implants conducted by residents may potentially elevate dental anxiety levels. Yet subsequent analysis of mDAS scores at T1 revealed a statistically significant reduction in anxiety levels among the healthy (ASA-PS I) patient group (*p* = 0.028).

Since dental anxiety is a multifactorial process, unique statistical analyses are needed to investigate its underlying causes, where we can examine the effect of different covariates. Furthermore, the use of repeated measurements in the same subject allows for a reduction in personality-related variables and the calculation of the reduction in dental anxiety. Certain studies mention the relative influence of patient personality traits for pain and dental anxiety [21]. Binary logistic regression analysis was used in this study to assess dental anxiety reduction (T0–T1), and the most appropriate model was determined by trying different covariates. Logistic regression analysis not only reveals statistical differences of covariates [62] but also assesses the likelihood of the predictors in reducing dental anxiety in the selected model [63]. Statistical analysis demonstrated that an improvement in the patient’s physical health status (ASA-PS I) decreases dental anxiety score (OR: 13.912). Conversely, the opposite was evident in patients with ASA-PS II and III.

Patients with ASA-PS II and III usually have comorbidities such as diabetes, hypertension, and arthritis that may lead to the need for postoperative analgesics [64], which may naturally be associated with less postoperative pain. It seems reasonable to posit that a reduction in pain is associated with a reduction in anxiety. A review of the literature shows that there is a positive correlation between dental anxiety and intraoperative pain perception [65,66]. Moreover, it was found that patients with higher dental anxiety scores showed more postoperative swelling 24, 48, 72 h, and even 1 week after surgery [67]. Notwithstanding, ASA-PS II and III (comorbid) patients showed only a slight decrease in mDAS scores, even though they showed less postoperative pain. The lack of a reduction in dental anxiety among patients with comorbidities cannot be attributed to alterations in pain perception. Therefore, ASA-PS may be regarded as a novel factor influencing dental anxiety. However, the fact that these patients have been under medical supervision for a long time due to their chronic diseases is also likely to diminish their anxiety. In fact, patients suffering from chronic systemic diseases have to face concerns about their illness and are subjected to medical interventions for diagnostic and therapeutic purposes [17]. Anxiety cannot, of course, be linked to physical health status alone. It is a complex process and depends on many variables.

A significant relationship was identified between age and reduction in the anxiety (OR: 1.089). The mean age of patients was 45.92 ± 11.65 years, with a median mDAS score at baseline of 11, indicating a moderate level of anxiety. Considering that most of these patients postpone their treatment, anxiety is naturally more pronounced in middle-aged groups (approximately one in three adults) [54]. Despite the absence of age group categorization, the findings of the present study exhibited similarities. The relationship between age (53.33 ± 8.42) and mDAS baseline became even more pronounced in the ASA-PS II and III (comorbid) patients. However, the results of a recent study indicated that mDAS scores of individuals aged 20–34 years were found to be higher than those of individuals aged 50–64 years [52]. In another survey with a large sample size, in which only the age variable was examined, it was reported that 16.4% of the participants had dental anxiety and only 27.1% of these patients experienced dental fear in adulthood [68]. The study mentioned that the effect of prior experiences, rather than age, was crucial. Other researchers also reported that dental anxiety decreases with age [43,69]. Since there is no consensus on the ‘Age’ variable, it is open to debate whether the difference is due to the increase in the frequency of systemic diseases that naturally develop with elderly or whether age is a separate determinant.

Another finding was that as the number of implants placed increased, there was a concomitant decrease in the reduction in dental anxiety (OR: 0.807). A total of 145 of 201 patients were treated with multiple implants. Although only a single quadrant was implanted in each session to avoid increasing anxiety, it increased in proportion to the increase in the number of implants. The number of implants has been linked to surgical complexity in certain studies, and anxiety scores demonstrate a tendency to increase in line with surgical complexity [16,53]. The SAC tool was employed to quantify surgical complexity in the present study. Patients with a treatment need outside of the conventional frame of SAC were excluded due to the necessity of general anesthesia for conditions such as iliac grafting and maxillofacial surgery. SAC appears to be a useful tool to assist dentists with less experience in implant dentistry in describing the complexity of the treatment [32]. Similarly, Abbas et al. employed the use of treatment complexity in conjunction with the mDAS score and ASA-PS as an assessment tool to ascertain the necessity for sedation [53]. Similarly, the surgical complexity associated with the extraction of deeply impacted third molars was found to be a contributing factor to dental anxiety [16]. Gülnahar et al. found that dental anxiety was positively correlated with the number of implants and demonstrated that systolic and diastolic blood pressure increased as the number of implants increased [70]. Bovaire et al. surprisingly found a negative correlation between anxiety and number of placed implants and attributed this result to the fact that patients with multiple implants are generally fearless [71]. From an alternative standpoint, research findings indicate that the quantity of implants may indirectly influence dental anxiety by affecting pain intensity [72]. In contrast, the number of implants was not identified an independent risk factor for moderate-to-severe postoperative pain [73].

The present findings indicate that ‘Gender’ being male increases the probability of anxiety reduction up to 6.493 times. The male participants exhibited considerably greater reductions in mDAS scores (Mann–Whitney-U test *p* < 0.001). In the study conducted by Muneer et al. [46], gender was found to be a significant variable at baseline anxiety. The study indicated that only 11% of male patients were identified as exhibiting phobic behavior, while this figure reached 20% in the female cohort. Similarly, Saba et al. demonstrated that women exhibited higher baseline anxiety scores. A lower median (9) mDAS score was found in this study, which is different from ours [52]. This notwithstanding, no significant difference was observed between genders in terms of baseline anxiety scores in our study. However, the mean mDAS scores at T1 were significantly higher for women. Similarly, a comparative study assessing pain following dental implant surgery [72] demonstrated that women exhibited significantly higher scores at the end of the first week (T1) compared to men. The literature also suggests that either there is no difference [74] or that women showed higher anxiety scores [75].

Although it was not found to be a significant variable in the present study, numerous other articles have found that dental anxiety is affected by socio-economic status and educational level [46,76]. However, there is also controversial evidence that individuals from lower socioeconomic backgrounds are more prone to dental anxiety [46]. Moreover, the likelihood of accessing misleading visual resources related to surgery is higher in patients with higher educational levels, and this may increase dental anxiety [77].

According to a recent study, prior surgeries seem to be a factor affecting dental anxiety. Patients with previous implant-supported prostheses exhibited statistically significantly lower anxiety and Oral Health Impact Profile-14 scores than patients with missing teeth and no previous implant surgery [13].

The most significant limitation of the study is that the data were collected in a single hospital setting, which limits their generalizability. Furthermore, the number of residents and specialists was not closely aligned, which may have resulted in change in patient anxiety levels. The inability to assess pain levels one day post-operatively hinders our ability to ascertain the progression of pain. Additionally, it is essential to consider that the selection of ASA-PS is predominantly influenced by the clinician’s expertise. Finally, the use of anxiolytic pharmaceuticals was not excluded as a potential factor contributing to elevated anxiety scores.

## 5. Conclusions

Within the limits of this this study, it was concluded that the self-assessment of mDAS scores was affected by the ASA-PS, which showed a greater anxiety reduction in healthy subjects (ASA-PS I) than in those with comorbidities (ASA-PS II and III). In addition to ASA-PS, gender and the number of placed implants affected the anxiety of the patients. Age and number of implants seem to be variables with a relative influence that depends on other factors such ASA-PS and surgical complexity. The findings are promising, but further research in a multicenter setting would be required to validate the results.

## Figures and Tables

**Table 1:** Variable description and their distribution.

Patient Demographics	ASA-PS I (*n* = 130)	ASA-PS II and III (*n* = 71)	*p* Value
*Comorbidities*			
No comorbidities	125		
Mild stomach problems	5		
Asthma		10	
Hypertension		33	
Hypothyroidism		4	
Type II diabetes		21	
Liver disease		12	
Rheumatoid arthritis		6	
Anticoagulant use		8	
*Age ^z^*	42.00 ± 11.23	53.33 ± 8.42	<0.001 **
*Gender*			0.30
Male	68 (52%)	31 (44%)	
Female	62 (48%)	40 (56%)	
*Experience*			0.73
Prior experience of implant surgery	73 (58%)	44 (62%)	
No experience of implant surgery	52 (42%)	27 (38%)	
*Dental Anxiety*			0.32
*Low Anxiety*	90 (69%)	56 (79%)	
Moderate Anxiety	25 (19%)	10 (14%)	
Severe Anxiety/Phobia	15 (12%)	5 (7%)	
**Dentist-related factors**			
*Experience*			0.002 *
Dentist experience (specialist)	32 (25%)	33 (46%)	
Dentist experience (resident)	98 (75%)	38 (54%)	
**Surgery-related factors**			
*Number of placed implants in the same session ^z^*	3.48 ± 2.11	3.23 ± 3.01	0.059
*Surgery time ^z^*	53.17 ± 30.80	78.90 ± 63.85	0.34
*Surgical complexity*			0.018 *
Straightforward surgery	86 (66%)	34 (48%)	
Advanced surgery	19 (15%)	21 (30%)	
Complex surgery	25 (19%)	16 (22%)	

* *p* < 0.05, ** *p* < 0.01 χ^2^: Chi-square test (categoric data), z: Mann–Whitney U *t*-test.

**Table 2:** Subjective self-assessment mDASs and NRSs change.

Patients		*p*
MDASs (T0)	11.57 ± 4.85	
Median (IQR)	11 (7)	<0.001 **
MDASs (T1)	10.26 ± 3.99	
Median (IQR)	11 (9)	
NRSs (T0)	5.26 ± 2.30	<0.001 **
Median (IQR)	5 (4)	
NRSs (T1)	3.88 ± 1.66	
Median (IQR)	4 (2)	

** *p* < 0.01; Wilcoxon-signed rank test; mDASs: Modified Dental Anxiety Scale Score. NRSs: Numerical Rating Scale for Pain Score.

**Table 3:** Subjective self-assessment of mDASs and NRSs according to study groups.

Patients	ASA-PS I	ASA-PS II and III	*p*
MDASs (T0)	11.53 ± 5.09	11.64 ± 4.40	0.563
Median (IQR)	11 (9)	12.5 (7.5)	
MDASs (T1)	9.63 ± 3.95	11.41 ± 3.85	0.007 **
Median (IQR)	10.50 (7)	11 (8)	
MDASs Reduction	1.77 ± 2.77	1.00 ± 1.88	0.028 *
Median (IQR)	1 (5)	0.5 (2.8)	
NRSs (T0)	5.30 ± 2.30	5.19 ± 2.33	0.741
Median (IQR)	5 (4)	5 (4)	
NRSs (T1)	3.86 ± 1.74	3.91 ± 1.51	0.373
Median (IQR)	3 (2)	5 (2)	
NRSs Reduction	1.46 ± 1.53	1.32 ± 1.56	0.573
Median (IQR)	2 (3)	1.5 (3)	

* *p* < 0.05, ** *p* < 0.01; Mann–Whitney-U test; mDASs T0: Modified Dental Anxiety Score at the day of surgery. mDASs T1: Modified Dental Anxiety Score one-week post-op. NRSs T0: Numerical Rating Scale for Pain score at the day of surgery. NRSs T1: Numerical Rating Scale for Pain score one-week post-op.

**Table 4:** Predictors in logistic regression model of dental anxiety reduction.

Variable	Explanation	β (Estimate)	Wald	Odds Ratio	*p*
Age	Continuous	0.085	8.882	0.003 **	1.089
Gender	Male	1.873	12.021	<0.001 **	6.493
ASA-PS	ASA-PS I	2.633	14.272	<0.001 **	13.912
Number of placed implants	Continuous	−0.214	3.810	0.051 *	0.807

0 = anxiety increased or not changed, 1 = anxiety reduced; * *p* < 0.05 ** *p* < 0.01 Hosmer and Lemeshow test, *p* = 0.60, Nagelkerke’s R^2^ = 0.370.

**Table 5:** Comparison of MDASs and NRSs according to gender.

Patients	Female	Male	*p*
MDASs (T0)	11.71 ± 4.65	11.42 ± 5.08	0.539
Median (IQR)	11 (7)	10 (6)	
MDASs (T1)	11.35 ± 3.93	9.17 ± 3.78	<0.001 **
Median (IQR)	11 (8)	10 (5)	
MDASs Reduction	0.765 ± 2.72	2.23 ± 2.05	<0.001 **
Median (IQR)	0 (2)	2 (2)	
NRSs (T0)	5.33 ± 2.32	5.19 ± 2.29	0.677
Median (IQR)	5 (4)	5 (4)	
NRSs (T1)	4.17 ± 1.62	3.58 ± 1.65	0.023 *
Median (IQR)	5 (2)	3 (2)	
NRSs Reduction	1.36 ± 1.63	1.47 ± 1.44	0.732
Median (IQR)	2 (3)	2 (3)	

* *p* < 0.05, ** *p* < 0.01; Mann–Whitney-U test; mDASs T0: Modified Dental Anxiety Score at the day of surgery. mDASs T1: Modified Dental Anxiety Score one-week post-op. NRSs T0: Numerical Rating Scale for Pain score at the day of surgery. NRSs T1: Numerical Rating Scale for Pain score one-week post-op.

**Figure A1:**
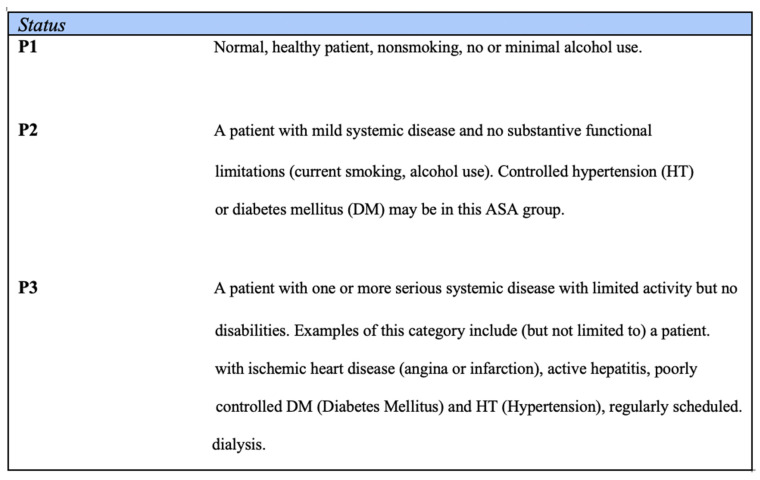
American Society of Anesthesiologists Physical Health Status Classification.

## Data Availability

The original contributions presented in this study are included in the article; further inquiries can be directed to the corresponding author.

## Appendix B

* Reliability and validity information see Ilguy et al. (2005) [33].

## Appendix C

* see McCaffery, M., Beebe, A. [78].
